# Effect of 5-Oxo-2-Pyrrolidinecarboxylic Acid (PCA) as a New Topically Applied Agent for Dry Eye Syndrome Treatment

**DOI:** 10.3390/pharmaceutics10030137

**Published:** 2018-08-25

**Authors:** Silvia Tampucci, Daniela Monti, Susi Burgalassi, Eleonora Terreni, Erica Zucchetti, Filippo Baldacci, Patrizia Chetoni

**Affiliations:** 1 Department of Pharmacy, University of Pisa, 56126 Pisa, Italy; silvia.tampucci@unipi.it (S.T.); daniela.monti@unipi.it (D.M.); eleonora.terreni@farm.unipi.it (E.T.); ericazeta93@gmail.com (E.Z.); patrizia.chetoni@unipi.it (P.C.); 2 Laboratori Baldacci S.p.A., 56100 Pisa, Italy; filippobaldacci@baldaccilab.com

**Keywords:** dry eye, hyaluronic acid, osmoprotectant, 5-oxo-2-pyrrolidinecarboxylic acid (PCA), rabbits, tear fluid

## Abstract

The aim of the study was the evaluation of the suitability of 5-oxo-2-pyrrolidinecarboxylic acid (PCA), also in combination with hyaluronic acid (HA), as artificial tears for treatment of dry eye syndrome (DES). Different aqueous formulations containing 0.10% *w/w* of PCA were used to determine: (i) ex vivo permeation profile of PCA in isolated rabbit corneas; (ii) in vivo residence time of PCA in the precorneal area of rabbits; and (iii) in vivo ability of PCA to counteract the reduction of tear production in an experimental model of DES induced in rabbits. The pharmacokinetic profile of PCA in tear fluid was characterized by high concentrations immediately after application, followed by a rapid decrease, with half-life values of 17.16 and 22.27 min for solutions containing PCA alone and in combination with HA, respectively, when 100 µL of solutions were instilled. The addition of HA almost doubled the PCA bioavailability minimizing the ex vivo apparent corneal permeability of PCA. A positive Shirmer Test Score (STS) was observed for PCA compared to contralateral eyes at all days of treatment for PCA/HA formulation. PCA provides protection from desiccation probably for its osmoprotective activity and high water–binding capability, and this behaviour was enhanced by HA.

## 1. Introduction

Dry eye syndrome (DES) is one of the most frequent ophthalmic pathologies and is generally characterized by an insufficient production or a scarce quality of tear secretion. This un-physiological condition causes a persistent dryness of the cornea and conjunctiva, as well as a keratoconjunctival disorder responsible for opacity of the cornea, anomalies in vision, and discomfort [1,2,3,4,5,6,7]. Dry eye syndrome has a number of causes, among which the more severe are vitamin A deficiency, Sjogren’s syndrome, rheumatologic diseases, chemical and thermal burns, or the use of specific drugs, such as hydrochlorothiazide, ketorolac, ketotifen, and levofloxacin [8].

One of the specific consequences of DES is hyperosmolarity of tears, a state in which the tear osmolarity is higher than that of intracellular fluids. Patients affected by DES have tear osmolarity values of 316–360 mOsmol/L with spikes up to 800–900 mOsmol/L that can be measured in the central part of the corneal surface, while normal osmolarity values are 285–295 mOsmol/L [9]. High values of osmolarity can be provoked by a reduced aqueous tear flow and/or an increase in the evaporation of the aqueous component of tear fluid, due essentially to a deficiency in the lipid constituent. Several morphological changes in conjunctival and corneal cells occur as result of the condition of hyperosmolarity, such as reduction of cell volume (cell shrinkage) and increase in the concentration of inorganic solutes. Other phenomena observed in presence of hyperosmolar conditions are the stimulation of the inflammatory cascade resulting in the release of mediators such as cytokines and proteolytic enzymes, the loss of both mucin producing goblet cells and the barrier function of the cornea, and even apoptosis of the ocular surface cells [10]. The use of hypotonic tear substitutes is the most common approach to reduce the ocular discomfort, even if these have a short residence time in the eye.

Recently, the use of osmoprotectants able to offset tear fluid hyperosmolarity has been evaluated as an advantageous strategy for DES treatment. Osmoprotection can be considered a natural reaction of the biological structures that causes, through accumulation of small biological molecules on cell surfaces, an adaptation of these structures to a hyperosmotic environment. An in vitro study reported decreased levels of mitogen-activated protein (MAP) kinases induced by hyperosmotic stress on human corneal epithelial cells in the presence of two osmoprotective agents, l-carnitine and erythritol [11]. A positive effect on cell viability in the presence of taurine supplementation was observed by Shioda et al. [12] that was ascribed to a possible antioxidant activity or membrane stabilization effect of taurine, a well-known osmolyte substance [13]. Osmoprotective substances such as erythritol, l-carnitine, and glycerine in combination with carboxymethylcellulose were more efficient in controlling primary outcomes (e.g., tear osmolarity, Schirmer-I test scores) and were consequently able to reduce the cellular stress level at the ocular surface in humans [14]. In addition, the ability of trehalose to allow for the survival of cells in unfavourable conditions, such as those present in a hyperosmotic environment, was exploited for use in the development of eye drops. The very high water retention capability of trehalose and its osmoprotective properties were effective in preventing cell death and improving the functionality of human corneal epithelial cells [15]. 

5-Oxo-2-pyrrolidinecarboxylic acid (PCA), known as pyroglutamic acid, is a small endogenous molecule chemically defined as the cyclic lactam of glutamic acid. It is derived from l-glutamate and represents the major intermediate of the *γ*-glutamyl cycle. The *γ*-glutamyl cycle is determinant for the synthesis and breakdown of glutathione and for the intracellular transport of amino acids [16]. The fundamental role of glutamate as a reservoir of pyroglutamic acid and of its parent molecule, glutathione, has been demonstrated in recent studies that have generally been performed on plants and in neuronal cells. However, the presence of 5-oxoprolinases in prokaryotic organisms that do not produce glutathione also suggests that it may have other biological functions. In particular, its possible role as an osmoprotective agent was studied in the halotolerant methanotroph (*Methylobacter alkaliphilum)*, during which pyroglutamic acid was found to accumulate in response to stress caused by an excessive concentration of salts. Furthermore, PCA is a natural component of mammalian tissue that is present in large amounts, especially in the horny layer of the skin. By virtue of the levels of PCA and PCA-generating enzymes in the skin, PCA has been proposed as a conditioning agent in cosmetic products, even if its absorption through skin is limited. In addition, PCA was proposed for ophthalmic use, both to promote the survival of axotomized retinal ganglion cells in adult mammals as mediated by excitatory amino acid transfer and to reduce intraocular pressure (IOP) in experimentally-induced ocular hypertension in rabbits [17].

In this context and in line with preliminary results [18], the purpose of the present study was to evaluate the suitability of the ophthalmic use of pyroglutamic acid as artificial tears for the treatment of DES. Eye drops containing PCA alone and in combination with hyaluronic acid, a well-known viscosifying and mucoadhesive polymer with great effectiveness in the preservation of corneal barrier function, were evaluated in rabbits by estimation of the residence time of PCA in tear fluid and determination of its efficacy in treating experimentally induced DES in rabbits. 

## 2. Materials and Methods

### 2.1. Chemicals

5-Oxo-2-pyrrolidinecarboxylic acid (PCA) was obtained from Usines Chimiques D’Ivry La Bataille (UCIB, Anet, France), and hyaluronic acid sodium salts (HA, MW 1.5–1.7 MDa) were manufactured by Contipro Biotech and supplied by Giusto Faravelli S.p.A (Milan, Italy). Sodium chloride, sodium phosphate dibasic monohydrate, monosodium phosphate dodecahydrate, disodium edetate dihydrate (EDTA), and sodium 1-hexanesulfonate were obtained from Sigma-Aldrich (Milan, Italy) and were used as received. Ultrapure water was prepared using a Milli-Q^®^ plus apparatus (Millipore, Milan, Italy). All other chemicals or solvents used were of reagent grade.

### 2.2. Animals

Female New Zealand albino rabbits weighing 2.8–3.5 kg were purchased from Pampaloni Rabbitry (Pisa, Italy). They were housed in standard cages in a light-controlled room (10 h dark/14 h light cycle) at 19 ± 1 °C with 50 ± 5% relative humidity and were given a standard pellet diet and water *ad libitum* [19]. During the experiments, the rabbits were placed in restraining boxes to which they had been habituated in a room with dim lighting, and they were allowed to move their heads and eyes freely in compliance with institutional guidelines and in accordance with the ARVO Statement for the Use of Animals in Ophthalmic and Vision Research. The research protocol was approved by the Committee for Animal Welfare of the University of Pisa and was authorized by the Italian Ministry of Health in compliance with the Legislative Decree 26/2014, which transposed the European Directive 2010/63/EU (the authorization number: 350/2018-PR; 9 May 2018; principal investigator D. Monti).

### 2.3. Preparation of 5-Oxo-2-Pyrrolidinecarboxylic Acid (PCA) Formulations

Three different experimental formulations (Formulations A, B and C) were prepared by dissolving an appropriate amount of PCA into ultrapure water using a magnetic stirrer in presence of sodium phosphate, EDTA and sodium chloride as isotonic agent. Hyaluronic acid (HA) was used as a viscosifying additive and mucoadhesive agent. The composition of the experimental preparations is shown in Table 1 together with their technological parameters. Sodium chloride ranging from 0.70–0.75% *w/w* was required to make the solutions isotonic (297–300 mOsmol/kg), as measured using a 5R Roebling Osmometer (Camlab, Cambridge, UK). The pH of all formulations was in the range of 6.6–6.8 (SevenCompact pH metre, Mettler Toledo, Milan, Italy).

Formulation A contained 0.10% of PCA and Formulation B contained 0.20% *w/w* of HA, while in Formulation C a mixture of 0.10% of PCA and 0.20% *w/w* of HA was used. An isotonic phosphate buffer solution was used as a reference (REF). All formulations were sterilized by filtration under laminar flow using hydrophilic PVDF membrane having a 0.22 µm pore size (SLGV004SL Filter Millex-GV, Millipore) and were used immediately after preparation. 

The evaluation of the viscosity of the formulations was performed at 30 °C using a Rheostress RS 1 Rheometer (Haake) equipped with coaxial cylinders (Z40 and Z41) at shear rates ranging from 0 to 200 s^−1^.

### 2.4. Ex Vivo Transcorneal Permeation of PCA

The ability of PCA to permeate through isolated rabbits corneas was examined using a perfusion apparatus in accordance with the method reported by Chetoni et al. [20]. The rabbits were euthanized with an intravenous lethal dose of pentobarbital (pentothal sodium, Farmaceutici Gellini, Aprilia, Italy) injected into the marginal ear vein after general anaesthesia was induced by i.m. injection of tiletamine hydrochloride (0.1 mg/kg Zoletil 50, Virbac srl, Milan, Italy). The eyes were rapidly proptosed, carefully washed with glutathione bicarbonate Ringer’s solution (GBR) and trimmed of all adventitial tissue. Then, corneas with a 2 mm ring of sclera were excised and mounted on a typical side-by-side diffusion cell in such a way that the epithelium faced the donor chamber. The corneal cross-sectional area that was available for diffusion was 0.79 cm^2^. Preheated GBR (35 °C) was added to both the epithelial (1.0 mL) and the endothelial (5.0 mL) compartment. The apparatus was maintained at 35 ± 1 °C and, to ensure oxygenation and agitation, an O_2_-CO_2_ (95:5) mixture was bubbled through each compartment at a rate of 3–4 bubbles per second [21]. After a pre-equilibration time of approximately 10 min, the GBR solution on the epithelial side was partially withdrawn and substituted with 1.0 mL of GBR solution containing 0.02% *w/w* of PCA (PCA) or 1.0 mL of a mixture of 0.02% *w/w* PCA plus 0.04% HA (PCA/HA). The concentration of PCA in the donor compartment was five times lower than that used in Formulations A and C, but the weight ratio between PCA and HA was maintained to be the same as that in Formulation C. In each case, the final concentration of PCA in the donor compartment was 0.20 mg. To ensure sink conditions, at appropriate time intervals 0.5 mL of the receptor solution was withdrawn for analysis and replaced with an equal volume of fresh preheated GBR buffer.

Each permeation experiment was continued for 4.0 h and was repeated six times. For analysis, each sample of the receiving phase (0.5 mL) was diluted with the mobile phase used for the HPLC method at a 1:1 volume ratio and analysed after centrifugation at 13,000 rpm for 10 min (IEC MicroCL 17, Thermo Electron Corporation, Waltham, MA, USA).

### 2.5. In Vivo Evaluation of the Precorneal Residence Time of PCA

The in vivo evaluation of the mean residence time of PCA in the precorneal area after application of Formulations A and C was carried out following the method reported by Stella et al. [19]. Six rabbits for each treatment group were positioned into restraining boxes and one drop (50 μL) or two drops (2 × 50 μL given at a 30 s interval) of both formulations, corresponding to 50 or 100 μg of PCA, respectively, were instilled into the lower conjunctival sac of one eye of each rabbit with a micro-syringe. In each case, the contralateral eye received the same amount of the reference solution (Table 1) in order to avoid experimental bias. At 1, 3, 5, 10, 20, 30, 40 and 60 min after administration, 1.0 µL of tear fluid sample was collected by a glass capillary (Drummond “Microcaps”, Fisher Scientific, St Louis, MO, USA) from the middle of the lower marginal tear, then transferred into a micro-vial and flushed with 1.0 µL of ultrapure water. After dilution with the mobile phase of the chromatographic HLPC method (2:58 volume ratio), tear fluid samples were centrifuged for 5 min at 13,000 rpm to precipitate the proteins and afterwards injected (20 μL) into the HPLC system. 

### 2.6. HPLC Analytical Methods

PCA concentration in the receiving phase of the ex vivo corneal permeation samples and in collected tear fluid samples from rabbits was measured using a Shimadzu LC-10AS apparatus equipped with LC-6AS pump, SPS-10AV detector, C-R4A integrating system, and 20 µL Rheodyne injector sample loop (Shimadzu Italia s.r.l., Milan, Italy). The mobile phase, delivered at a flow rate of 1.0 mL/min in a 10 µm reversed-phase C_18_ column (Bondclone 30 mm × 3.9 mm i.d., Phenomenex, Torrance, CA, USA), was a mixture of a 98:2 ratio of buffer solution and acetonitrile. The buffer solution consisted of 4.3 mM chloride acid and 0.1 mM sodium 1-hexanesulfonate. The detection wavelength was 210 nm and the retention time of PCA under these conditions was 2.98 min. The amount of PCA in the samples was determined by comparison with an external standard curve obtained by dilution with the mobile phase of different aqueous solutions of PCA in water. The calibration curve was obtained by applying least square linear regression analysis to experimental data obtained for PCA at a concentration ranging from 0.20 to 10.06 µg/mL by using Prism 7 software (GraphPad Software Inc., San Diego, CA, USA). The correlation coefficient calculated for the experimental data points was 0.9992 (*R*^2^) and the limit of detection (LOD) was 0.08 µg/mL.

### 2.7. Treatment of the Experimental Condition of Dry Eye in Rabbits

The animals were preliminarily examined to confirm the integrity of the corneal surface and to exclude the presence of ocular disorders that would modify the lacrimal function, such as dryness. The condition of the corneal surface was evaluated by visual analysis with a slit-lamp after application of an isotonic aqueous solution of 0.5% *w/v* sodium fluorescein (10 µL), while the amount of tears produced in physiological conditions was detected by using the Shirmer Test I. For this test, paper test strips (Alfa Intes, Casoria, Italy) were placed into the lower conjunctival fornix of each of the rabbit eyes and maintained there for 3 min. At least 5.0 mm of paper was required to be soaked by tears to exclude any condition of ocular dryness and to enrol the animals in the treatment groups. The selected rabbits were divided into three different groups, each consisting of 6 animals, and all of the rabbits were treated according to the experimental protocol reported by Burgalassi et al. [22,23] in order to induce the condition of dry eye. Briefly, the animals were given 50 µL of 1.0% *w/v* atropine sulphate aqueous solution (AS, Atropine 1%, Farmigea SpA, Pisa, Italy) in the lower conjunctival sac of both eyes at 9:00 a.m., 1:00 p.m. and 5:00 p.m. Five minutes after this treatment, they received 100 µL (two drops of 50 µL given at a 30 s interval) of the formulations being studied (Formulations A, B and C) in the left eye and the same amount (two drops of 50 µL given at a 30 s interval) of the reference solution in the right eye (Table 1). All treatments were performed continuously for five days from Monday to Friday. The lacrimation produced during the treatment was evaluated the first day, immediately before the first AS instillation (*X*_0_, 1st day) and subsequently at the second, third, fourth and fifth day after AS instillation (10:00 a.m.) by placing test strips into the lower conjunctival fornix of both eyes of each rabbit for 3 min (*X*_t_). The wetted length of each strip was measured in millimetres and the result was reported as a Shirmer Test Score (STS), defined as the difference between *X*_t_ and *X*_0_ (Δ(*X*_t_ − *X*_0_)). STS values were calculated for each eye and the data are expressed as the mean ± SE.

### 2.8. Evaluation of Corneal Integrity

The corneal surfaces of different groups of treated rabbits were observed with a slit-lamp biomicroscope fitted with a blue filter after staining with fluorescein ophthalmic strips (Fluorets^®^ 1 mg, Bausch & Lomb, Kingston Upon Thames, UK). The strips were applied into the lower fornix, according to the manufacturer’s instructions, and after 3 min the eyes were photographed. The evaluation was carried out at 2:00 p.m. on the third, fourth and fifth days before the administration of AS. Dry dots on the corneal surfaces of the rabbits were considered a symptom of corneal dehydration and were revealed by the presence of fluorescein punctate stains on the corneal surfaces [23]. 

### 2.9. Pharmacokinetics and Statistical Analysis

The apparent corneal permeability coefficients (*P*_app_) of PCA were calculated from the slope of the steady state from the linear plots of the amount of PCA permeated through the isolated corneas in the receiving chamber (Q) versus time (*t*) during the ex vivo corneal permeation study. The *P*_app_ was defined by the equation *P*_app_ = ∆*Q*/(∆*t* × *C*_0_ × *A*), where *A* is the corneal surface in contact with the donor phase and *C*_0_ is the concentration of PCA in the donor phase at the beginning of the permeation experiment.

Linear regression analyses (slopes and correlation coefficients) were performed using Prism 7 software(GraphPad Software Inc., San Diego, CA, USA).

The area under the PCA concentration in tear fluid versus time curves (AUC) was calculated from the first observation (1 min after instillation, *t*_1min_) to the last observation point, using appropriate graphs and applying the linear trapezoidal rule. The apparent first-order elimination rate constants of PCA from tear fluid (*K*e_tf_) and the corresponding half-lives (*t*_1/2_) were calculated from the log-linear phase of drug concentration versus time profiles (range: 5 min—end point) [19]. 

Statistical differences between PCA concentrations at the different times of sampling (i) in the receiving phase during the ex vivo corneal permeation test and (ii) in tear fluid after instillation of Formulation A and Formulation C were evaluated using Student’s two-tailed unpaired *t*-test (Prism 7 software). The evaluation included calculation of the mean and standard error (S.E). Differences were considered statistically significant at *p* < 0.05. 

For the dry eye rabbit model, the differences among the experimental groups of rabbits treated with the formulations were subjected to statistical evaluation using one-way ANOVA analysis (Prism 7 software). The differences were considered significant at *p* < 0.05.

## 3. Results

### 3.1. Ex Vivo Permeation of PCA through Isolated Rabbit Corneas

**Figure 1:**
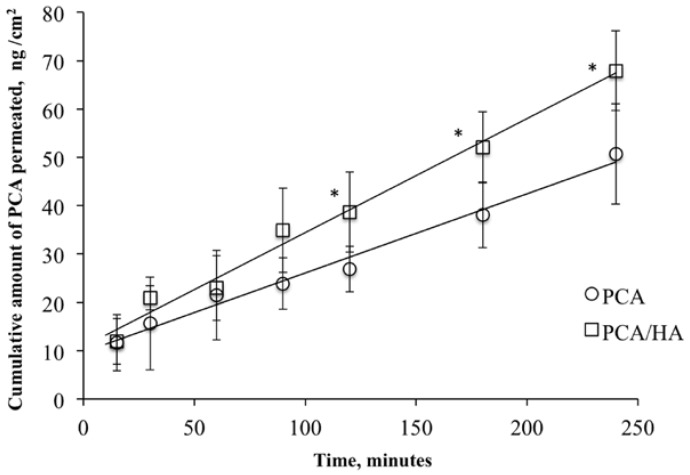
Permeation profile of 5-oxo-2-pyrrolidinecarboxylic acid (PCA) across isolated rabbit corneas for PCA (○) and PCA/HA mixture (□). Means ± SD; *n* = 6. * Statistically significant different, (*p* < 0.05, *t*-test).

The permeation profile of PCA through isolated rabbit corneas is reported in Figure 1 as cumulative amounts of PCA permeated versus time. The experimental data for the cumulative amount of PCA detected in the receiving chamber (*Q*) versus time (*t*) at the steady-state were highly correlated (*R*^2^ = 0.9854 and 0.9870 for PCA and PCA/HA, respectively), demonstrating the integrity of the corneal barrier during all of the permeation experiments. The flux values (*J*) at the steady-state, calculated from the linear ascents of the permeation graph, were 1.64 × 10^−4^ µg cm^−2^ min^−1^ for PCA and 2.36 × 10^−4^ µg cm^−2^ min^−1^ for the PCA/HA mixture (Table 2). Corneal permeability is an intrinsic parameter and defines the capability of a molecule to diffuse across the tissue, independent of the apparatus and/or protocol chosen for the experiment. PCA apparent corneal permeability (*P*_app_) was 1.972 × 10^−8^ and 1.361 × 10^−8^ cm s^−1^ for the PCA and PCA/HA preparations, respectively. The results indicated that PCA permeated the corneal tissue at the same order of magnitude for both PCA and PCA/HA solutions. The fluxes of PCA through the isolated corneas were not significantly different for the two preparations (*p* > 0.05, *t*-test), however the presence of hyaluronic acid seems to induce an appreciable increase in the affinity of PCA for corneal structures, favouring its corneal permeation. Indeed, statistically significant differences (*p* < 0.05, *t*-test) were obtained for the amount of PCA permeated through the cornea after 120, 150, and 240 min when the PCA and HA mixture was used as the donor phase, compared to that obtained with PCA alone. 

### 3.2. Precorneal Pharmacokinetics

**Figure 2:**
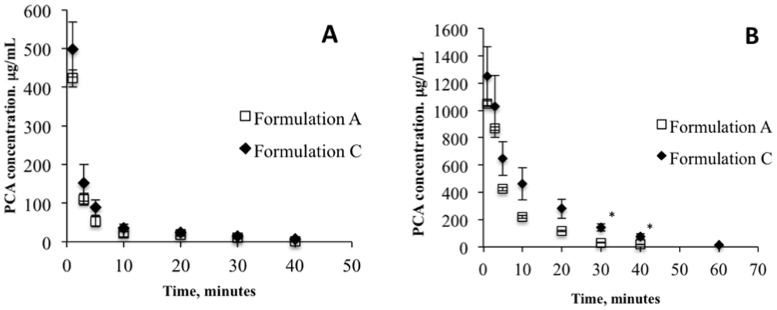
Concentration of PCA in tear fluid of rabbits after administration of 50 µL (**A**) and 100 µL (**B**) of the formulations under study. Means ± SE, *n* = 6. * Statistically significant different, (*p* < 0.05, *t*-test).

Formulations A and C, containing the PCA alone and the PCA-hyaluronic acid mixture, respectively, were well tolerated in vivo. Any symptoms of ocular inflammation or irritation were observed in rabbits for both of the applied doses by using a modified Draize test (unreported data). The precorneal PCA concentration versus time profiles obtained for both formulations (A and C) after application to the conjunctival sac of rabbits of different doses are reported in Figure 2. The relevant pharmacokinetic parameters calculated from the PCA concentration versus time curves are reported in Table 3 and were: (i) the area under the curve; (ii) the apparent first order elimination rate constants from tear fluid (*K*e_tf_); and (iii) the half-life of elimination of the drug from the precorneal area (*t*_1/2_). The pharmacokinetic profiles were characterized by high PCA concentrations in tear fluid immediately after instillation, followed by a rapid decrease in concentration 5 min after the application of both 50 and 100 µL of Formulations A and C. 

The precorneal elimination of PCA was well illustrated, with a mono-exponential equation with apparent first-order elimination rate constants of 4.19 × 10^−2^ and 3.34 × 10^−2^ min^−1^ and half-life values of 16.59 and 20.75 min for Preparation A and Preparation C, respectively, at the applied dose of 50 µL (*R*^2^ = 0.9965–0.9955). Superposed elimination rate constants were calculated at the higher dose (100 µL), with *K*e_tf_ values of 4.05 × 10^−2^ and 3.12 × 10^−2^ min^−1^ and corresponding *t*_1/2_ values of 17.16 and 22.27 for Formulations A and C, respectively. As evidenced by the ratios between AUC values, the addition of the mucoadhesive polymer (HA) to the aqueous PCA formulation (Formulation C) increased the bioavailability of PCA in tear fluid, up to 1.41 and 1.9 times for 50 and 100 µL of applied volume, respectively. Furthermore, a significant improvement in PCA bioavailability of 5.7- and 7.9-fold was obtained by increasing the applied volume from 50 to 100 µL for Formulation A and Formulation C, respectively. In conclusion, PCA bioavailability in tear fluid was improved by both the instillation of a higher volume and in the presence of HA.

Generally, the residence time of active ingredients in the conjunctival sac of the eye is relatively short, usually less than 1–2 min due to the permanent production of tear fluid (0.5–2.2 µL/min) [24] and the rapid and extensive drainage of the applied formulations from the precorneal area. The presence of PCA in conjunctival sac for a long time, up to 40 min, highlights its affinity for ocular structures. Furthermore, the bioavailability of PCA in tear fluid increased in the presence of HA which lead to a higher viscosity value for the formulation (13.5 mPa·s), as well as increased mucoadhesiveness. Hyaluronic acid is able to adhere to the ocular surface due to its biochemical properties and/or physical mechanism and binds to the CD44 cell surface adhesion molecule, which has been found on corneal epithelial cells [25]. Furthermore, the ability of HA to create a gel network with its mobile and flexible chains facilitates an interdiffusive process in the depths of the mucus layer. These behaviours became more appreciable with repeated application, such as the use of two drops of Formulation C within 30 s. Despite the fact that the elimination rate constant was independent of the applied dose (4.19 and 4.05 × 10^2^ min^−1^ for 50 and 100 µL of Formulation A, respectively; and 3.34 and 3.12 × 10^2^ min^−1^ for 50 and 100 µL of Formulation C, respectively), the higher PCA concentrations detected in the tear fluid 30 and 40 min after application (*p* < 0.05) highlighted the efficacy of hyaluronic acid in prolonging the presence of PCA in the precorneal area, especially immediately after application.

### 3.3. Activity of the Formulations in the Dry Eye Rabbit Model

**Figure 3:**
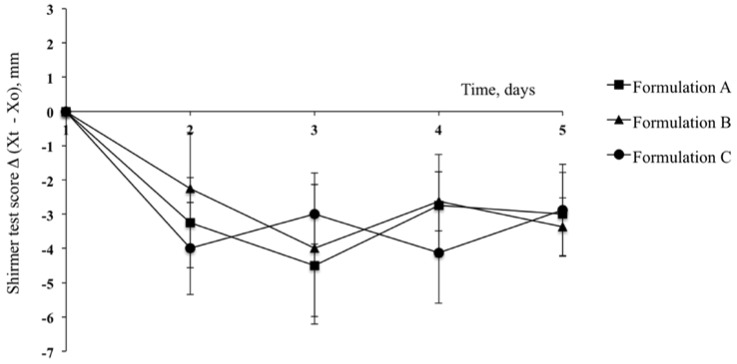
Shirmer Test Score for the contralateral eyes (Reference) after treatment with atropine sulphate aqueous solution (AS) for the different treatment groups. Means ± SE.

A noteworthy reduction in tear fluid production in albino rabbits was detected in the contralateral eyes of rabbits from all of the different groups that had received the reference solution exclusively. The difference in tear fluid production at the different time points (*X*_t_) with respect to the basal lacrimation (*X*_0_) are reported for the contralateral eyes in Figure 3 as Shirmer Test Scores (STS = *X*_t_ − *X*_0_). The STSs were in the range of −2.0/−4.5 mm from the second day up to the end of treatment, with a maximal value equal to −4.5 ± 1.49 mm for the group of rabbits treated with PCA on the third day. The results of the ANOVA statistical test confirmed that the STSs for the contralateral eyes for all treatments on the second, third, fourth and fifth days were significantly different from the corresponding basal values (first day); however, there were no statistical differences among them (*p* > 0.05, ANOVA). These results confirmed the efficacy of the treatment with AS in reducing tear fluid production and demonstrated that this method was suitable for the biopharmaceutical evaluation of ophthalmic products. The significant reduction of aqueous production after AS administration is due to its antimuscarinic activity in the lacrimal gland, with evident modification of tear stability [26]. This protocol leads to the development of dry-eye syndrome in rabbits and was applied to the evaluation of the efficacy of new polymeric substances such as arabinogalactan [27], and of human natural constituents such as 2-fucosyl-lactose [28]. 

**Figure 4:**
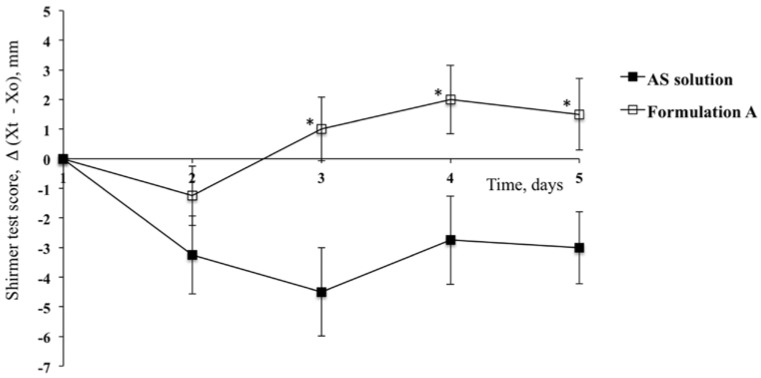
Shirmer Test Scores for the group of rabbits treated with Formulation A and for contralateral eyes (Reference) after treatment with AS solution. (Means ± SE, *n* = 6). * Statistically significant different from the STS calculated for the contralateral eye (*p* < 0.05, *t*-test).

An important contrasting effect on induced dry-eye syndrome was observed for Formulation A, which contained PCA alone (Figure 4), whereupon a slight counteraction of the effect of atropine on the second day of treatment and a more considerable effect on the third day of application was detected. In fact, the STS was −1.25 compared with −3.25 for the contralateral eye on the second day and was positive (STS = 1.0) after the third day of treatment, varying between 1.5 and 2.0 mm up to the fifth day. In any case, statistically significant differences (*p* < 0.05, *t*-test) were obtained for all time points after application of Formulation A when compared to that of the contralateral eye.

**Figure 5:**
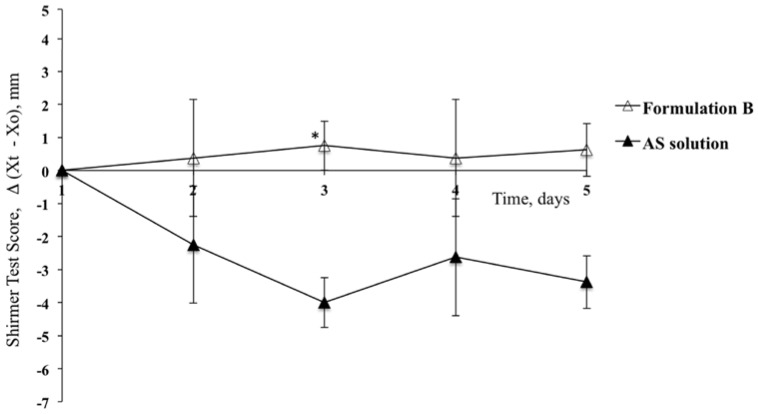
Shirmer Test Scores for the group of rabbits treated with Formulation B and for contralateral eyes (Reference) after treatment with AS solution. (Means ± SE, *n* = 6). * Statistically significant different from the STS calculated for the contralateral eye (*p* < 0.05, *t*-test).

A lower efficacy was observed upon the application of Formulation B, containing hyaluronic acid alone (Figure 5), where a constant counteraction of the reduction of produced tear fluid was detected, but with statistically significant differences found with respect to comparison with the contralateral eye exclusively on the third day of treatment (STS = 0.75; *p* < 0.05, *t*-test). 

**Figure 6:**
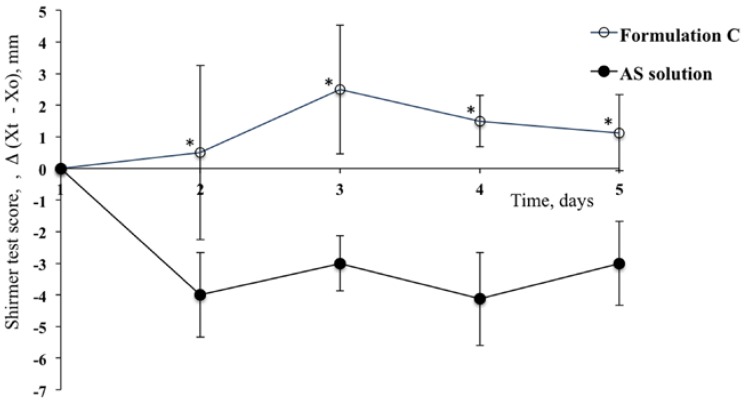
Shirmer Test Scores for the group of rabbits treated with Formulation C and for contralateral eyes (Reference) after treatment with AS solution. (Means ± SE, *n* = 6). * Statistically significant different from the STS calculated for the contralateral eye (*p* < 0.05, *t*-test).

The application of Formulation C, containing a mixture of PCA and HA, produced a remarkable reduction of the lacrimation induced by AS, and this activity was considerable even after only one day of treatment. In fact, a positive STS was obtained for Formulation C on the second day of treatment (STS = 0.5) and this increased on the third day (STS = 2.5). This activity was higher also when compared to that obtained for Formulation A (third day: STS = 2.5 and 1.0 for Formulation C and Formulation A, respectively). In any case, the Shirmer test values were significantly different for all days of treatment for Formulation C when compared to those obtained for the contralateral eyes that treated with the reference solution (Figure 6; *p* < 0.05, *t*-test).

These results have demonstrated the efficacy of PCA when used in combination with the mucoadhesive HA polymer to prevent atropine-induced reduction of tear volume in rabbits. Furthermore, the association of PCA and HA lead to an improvement of the Shirmer test values within the first few minutes after application.

### 3.4. Corneal Integrity after Treatment with the PCA Formulations

**Figure 7:**
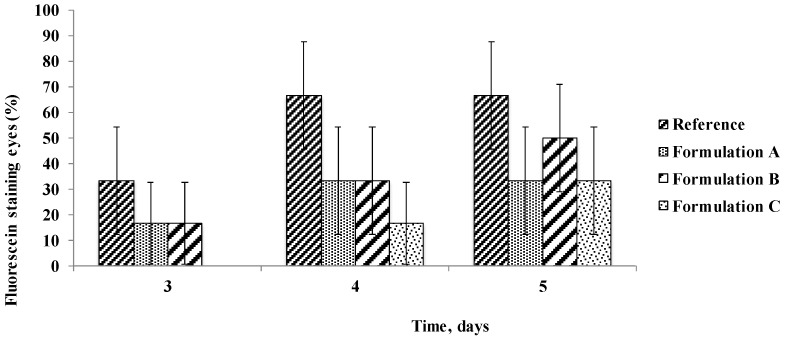
Percentage of fluorescein staining eyes during time in response to AS solution treatment in the contralateral eyes (Reference) and in the eyes treated with the Formulations under study after instillation of AS solution (Means ± SE, *n* = 6).

**Figure 8:**
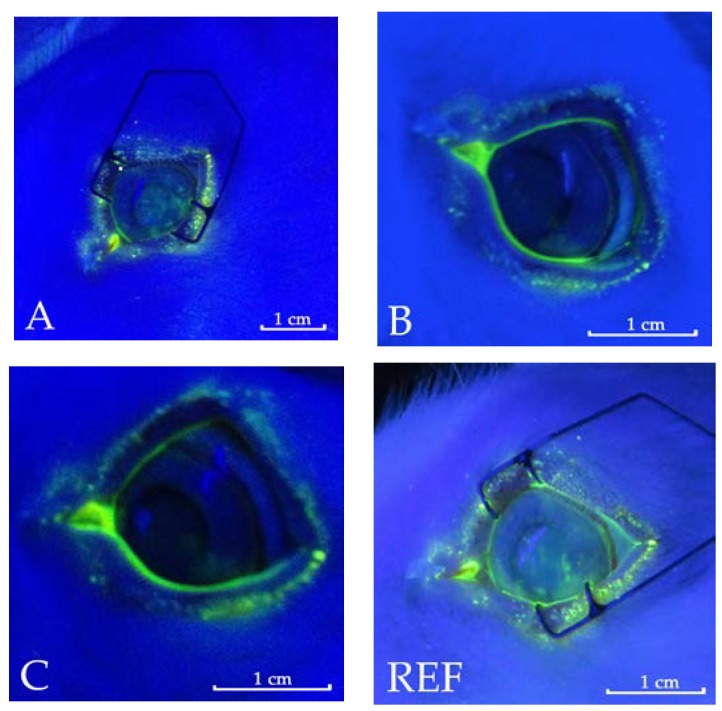
Representative corneal images for the eyes treated with Formulation (**A**), (**B**), (**C**), and (**REF**) at 4 days. For REF and (**A**) the images are taken for the fluo-positive eyes while for (**B**) and (**C**) the eyes are without coloured spot on the cornea.

In the present study, corneal staining that represented an important signifier of DES and ocular surface irritation was reduced by administration of all the formulations under study upon comparison of the eyes treated with the Reference solution. The use of PCA-based eye drops, favouring osmoprotection of the epithelial corneal cells, can reduce desiccation of the ocular tissues. This result was assessed by observation with a slit lamp equipped with a blue filter after staining the cornea with fluorescein, expressed as the percentage of eyes showing dotted staining on the third, fourth and fifth days of treatment. The percentage of the eyes with stained area on the corneal surface (fluo-positive eyes) was 30–66% for the control eyes treated with the Reference solution and up to 50% after administration of Formulation B, containing HA, at the different times of treatment (Figure 7). On the other hand, a limited incidence of fluorescein staining was observed on rabbit corneas after instillation of the Formulations A and C based on PCA, for which the maximal value of fluo-positive eyes was 33%. In particular, no fluo-positive eyes were found on the third day of treatment. In any case, the percentage of eyes with dry spots after treatment with PCA based Formulations was always 50% of value obtained for the control eyes. As an example, pictures of the eyes with and without stained spots were reported for the different treatments at 4 days in Figure 8.

## 4. Discussion

The results of the present study suggest that PCA might be effective for use in eye drops for the treatment of DES. In the last two decades, several strategies have been proposed for dry eye management as a function of disease severity, due to the increased knowledge regarding its aetiology and physiopathology. Indeed, the ocular systems involved in DES consist of the ocular barriers and ocular annexes, such as the cornea, conjunctiva, lacrimal glands, meibomian glands, nasolacrimal duct, as well as the eyelids and eyelashes. Furthermore, the functionality of the eye depends upon a complex system of relationships, where the nervous system’s apparatus of transmission and the endocrine, immune, and vascular systems contribute to the regulation of the functionality of this organ. Under these circumstances, many treatment options among those currently available might be chosen to manage DES. 

The maintenance of tear film stability and the reduction of tear hyperosmolarity in regard to all consequent symptoms, including corneal inflammation, are generally crucial factors chosen as therapeutic targets [29,30]. Tear replacement, often with hypotonic ocular lubricants, is traditionally considered the first-level treatment for DES therapy, and numerous topical formulations are available. Artificial tears, generally based on water-retentive polymers such as carboxymethylcellulose and hyaluronic acid, replace and/or reproduce the natural tear film and control the inflammatory response [31,32,33]. An improvement of the efficacy of tear substitutes can be obtained with the addition of osmoprotective agents that contribute to the stabilization of the cell volume under hyperosmotic stress, limiting cellular apoptotic phenomena and inflammation processes [14].

‪Notwithstanding an update to the knowledge of the pathophysiology of dry eye in the last few years, the request for satisfactory treatment of DES encourages the development of new ingredients. Our findings showed that the hypotheses regarding the selection of new osmoprotective ingredients for the treatment of DES should be pursued. PCA was selected as a small endogenous molecule that was able to meet expectations of demonstrating advantageous behaviour to decrease DES symptomatology. 

It is known that alterations of the secretion system at the eye surface produce changes in the tear film or corneal epithelial surface (e.g., tear volume and osmolarity), making the corneal epithelium susceptible to desiccation and damage. Several animal models have been proposed to evaluate the influence of the different ocular structures to occurrence of this pathology and to develop new treatments. In any case, one of the main symptoms common to all types of models of dry eye is the scarce tear fluid production and all the different experimental animal models carry out measurements of aqueous tear production using Shirmer test. In the atropine-induced dry eye model in rabbits, other than a reduction of the Shirmer test score to 8.75 mm, an improvement of approximately 93.0 mOsmol/L was observed in the tear fluid of rabbits after 24 h of treatment [28].

In our study, the scarce tear volume in contact with the corneal epithelial cells in atropine-induced dry eye in rabbits was affected by the presence of hydrophilic PCA, which was able to provide protection from desiccation by way of its osmoprotective activity and high water-binding capability and thereby maintain a significant tear volume in the conjunctival sac [16]. Furthermore, the combination of PCA and HA allows for a significant improvement in this behaviour probably due to the capability of HA to hold approximately 1000 times the weight in water respect to the surrounding tissue [34]. In addition, even though corneal staining was reduced by administration of all the formulations studied, upon comparison of atropine-treated and Reference solution-treated eyes, only Formulations A and C, which both contained PCA, were able to considerably reduce the number of fluorescein-stained eyes, thus confirming their efficacy. Interestingly, even if the interpenetration of hyaluronic acid chains with the mucus layer covering the cornea can entrap the PCA molecule on the eye surface, favouring its efficacy, transcorneal permeation does not occur. A similar behaviour was observed by Ito et al. [17] during an ex vivo PCA corneal permeation study using eye drops containing promotion enhancers such as cyclodextrins; they found a substantially higher PCA transcorneal permeation, with a flux of 10.52 nmol cm^−2^ h^−1^, by using a five-fold higher PCA concentration in the donor compartment of the perfusion apparatus. This flux value was approximately 100-fold higher with respect to the values of 0.0761 and 0.1096 nmol cm^−2^ h^−1^ measured in our study for PCA alone and the PCA/HA mixture, respectively. These results should exclude the effect of PCA in reducing intraocular pressure for the formulations studied and, therefore, allow them to be considered safe for use as eye drops.

## 5. Conclusions

Our results demonstrate that PCA is effective as a component of eye drop formulations for the treatment of DES. The combination of PCA, as an osmoprotective ingredient, and HA, as a pharmaceutical mucoadhesive and viscosifying substance able to optimize the ocular pharmacokinetic behaviour of PCA, may be the more promising combination for use in the treatment of DES. 

## 6. Patents

Baldacci M. Pyrrolidone carboxylic acid (PCA) for ophthalmic use. WO2016/162759A1.

## Figures and Tables

**Table 1:** Composition (% *w/w*) and technological parameters of the formulations under study.

Preparations	PCA	HA	Na_2_HPO_4_ 12H_2_O	NaH_2_PO_4_ H_2_O	EDTA 2H_2_O	pH	Osmolality (mOsmol/Kg)	Viscosity (mPa·s)
Formulation A	0.10	-	0.60	0.06	0.05	6.8	297	1.5
Formulation B	-	0.20	0.60	0.06	0.05	6.6	300	12.9
Formulation C	0.10	0.20	0.60	0.06	0.05	6.7	298	13.5
Reference	-	-	0.60	0.06	0.05	6.9	299	1.2

**Table 2:** Pharmacokinetic parameters for the ex vivo transcorneal permeation of -Oxo-2-pyrrolidinecarboxylic acid (PCA). Means ± SE, *n* = 6.

Preparations	Flux 10^4^ µg cm^−2^ min^−1^	*P*_app_ 10^8^ cm/s	PCA Permeated * % *w/w*
PCA	1.64 ± 0.30	1.972 ± 0.25	0.049 ± 0.001
PCA/HA	2.36 ± 0.23	1.361 ± 0.19	0.051 ± 0.002

* cumulative amounts of PCA permeated through rabbits corneas.

**Table 3:** Pharmacokinetic parameters in tear fluid after topical administration of the formulations under study. Means ± SE, *n* = 6.

Preparations	Instilled Volume (µL)	*C*_1min_ (µg mL^−1^)	*K*e_tf_ (10^2^ min^−1^)	*t*_1/2_ (min)	AUC (min µg mL^−1^)
Formulation A	50	422.5.0 ± 44.9	4.19 ± 0.81	16.59 ± 0.84	1310 ± 385
	100	497.4 ± 163.4	4.05 ± 0.79	17.16 ± 1.42	7476 ± 1674
Formulation C	50	1049.5 ± 86.4	3.34 ± 0.85	20.75 ± 2,33	1854 ± 432
	100	1250.0 ± 419.3	3.12 ± 0.66	22.27 ± 1.21	14576 ± 5655
